# Supplementary material for: γ-Aminobutyric Acid Priming Alleviates Acid-Aluminum Toxicity to Creeping Bentgrass by Regulating Metabolic Homeostasis
Source: Int J Mol Sci. 2023 Sep 20;24(18):14309. doi: 10.3390/ijms241814309 (PMC10532299; doi:10.3390/ijms241814309)
Supplement: Supplementary file 1 [file ijms-24-14309-s001.zip › ijms-2597039-supplementary.pdf]

Table S1 Relative retention time and mass to charge ratios of 73 identified metabolites in leaves of creeping bentgrass.

NO.	RT (min)	Metabolites	m/z	NO.	RT (min)	Metabolites	m/z
1	3.726	Oxalic acid	150	38	18.661	Glutamic acid	246
2	4.568	Pyruvic acid	174	39	19.686	Levoglucosan	204
3	4.738	Lactic acid	117	40	20.084	Lyxose	452
4	4.816	Oxamic acid	190	41	20.597	Ribose	307
5	5.006	Glycolic acid	66	42	20.836	Glucose	307
6	5.281	Valine	146	43	21.897	Galactose	346
7	5.494	Alanine	116	44	21.992	Xylitol	307
8	6.554	Norleucine	86	45	22.142	Ribitol	307
9	7.004	Isoleucine	86	46	22.335	Putrescine	174
10	7.096	Succinate semialdehyde	142	47	23.535	Gluconic lactone	230
11	8.479	Urea	192	48	23.605	Glucose-1-phosphate	450
12	9.110	Leucine	158	49	24.514	Azelaic acid	317
13	9.142	Glycerol	205	50	24.673	Saccharic acid	333
14	9.319	Cycloleucine	195	51	25.180	Shikimic acid	357
15	9.680	Proline	142	52	25.290	Citric acid	273
16	9.815	Maleic acid	245	53	25.985	Dehydroascorbic acid	316
17	9.880	Glycine	147	54	26.542	Quinic acid	345
18	10.094	Succinic acid	247	55	26.995	Sorbose	470
19	10.364	6-Hydroxy caproic acid	261	56	27.612	Talose	319
20	10.452	Glyceric acid	292	57	27.664	Beta-mannosylglycerate	204
21	10.982	Fumaric acid	245	58	28.583	Lysine	317
22	11.195	Serine	278	59	29.000	Mannitol	293
23	11.803	Threonine	291	60	29.013	Tyrosine	280
24	13.263	Glutamine	156	61	29.303	Ascorbate	332
25	13.878	Citramalic acid	247	62	29.691	Maltose	361
26	14.138	Erythrose	234	63	30.966	Gluconic acid	333
27	14.426	Malic acid	233	64	33.691	Myo-inositol	305
28	14.750	Asparagine	100	65	37.280	Linoleic acid	337
29	15.018	Threitol	307	66	40.403	Glucose-6-phosphate	387
30	15.103	Dithioerythritol	323	67	40.448	Galactinol	337
31	15.300	Oxoproline	156	68	42.794	Trehalose-6-phosphate	318
32	15.416	Aspartic acid	232	69	45.211	Maltotriitol	361
33	15.705	γ-Aminobutyric acid	294	70	47.857	Sucrose	361
34	15.801	Maleamate	346	71	49.502	Gentiobiose	160
35	16.675	Threonic acid	292	72	56.929	Kestose	289
36	16.969	Aconitic acid	229	73	58.869	Raffinose	204
37	17.164	Alpha-ketoglutaric acid	198				

Table S2 Details of primer sequences of tested genes.

Target gene	Gene accession number	Forward Primer (5′-3′)	Reverse Primer (5′-3′)	TM (°C)
ALMT9-like	OQ773408	GGTGGCCATGCTGGTATTA	GCCTTCGCTTCAACAACTTC	62
STOP1-like	OQ773409	GCCACTGTGACAAGAGCTATAC	CCCAAAGAGCTTGTCCTTTCT	62
MATE12-like	OQ773410	TGAGGAGGAGGTCATAAGT	TCCCGACAAGATAGAACG	62
MATE14-like	OQ773411	ATCAGCATATCCTACTGGGTCA	CGCAAGAACACGCTCACTC	62
MATE27-like	OQ773412	GTGAGGGTGGCAAACGAG	TTGTATGCGAGGATGAGGC	62
MATE29-like	OQ773413	CTTCAGCGACAACGACGAG	GGGAATGCCCACCAAATAG	62
MATE48-like	OQ773414	AACAACGCCTCCTCCATTC	GCAAGCCTTTCTCTCTCTCTTT	62
CS-like	OQ773415	CGAAGCAGGTTAGGGATAAA	TATTTGAAGGCAGGACAACA	62
cMDH-like	OQ773416	CCAGGAAGGAAGGAATGGAAA	CTCAGGGATAGATGGAGCAAAC	62
mMDH-like	OQ773417	CTCACCAAGAGGACACAAGATG	GCACAAAGGAGCACTCGATTA	62
3-cIPMDH 2-like	OQ917531	CCAGTTGTTGGTGGTCAT	CCCGCATTCTGTATCCTCTT	62
Actin	DY543529	CCTTTTCCAGCCATCTTTCA	GAGGTCCTTCCTGATATCCA	62
